# A Stochastic Simulation Framework for the Prediction of Strategic Noise Mapping and Occupational Noise Exposure Using the Random Walk Approach

**DOI:** 10.1371/journal.pone.0120667

**Published:** 2015-04-15

**Authors:** Lim Ming Han, Zaiton Haron, Khairulzan Yahya, Suhaimi Abu Bakar, Mohamad Ngasri Dimon

**Affiliations:** 1 Faculty of Civil Engineering, Universiti Teknologi Malaysia, Skudai, Malaysia; 2 Faculty of Electrical Engineering, Universiti Teknologi Malaysia, Skudai, Malaysia; Centre for Computational Biology and Evolution, CHINA

## Abstract

Strategic noise mapping provides important information for noise impact assessment and noise abatement. However, producing reliable strategic noise mapping in a dynamic, complex working environment is difficult. This study proposes the implementation of the random walk approach as a new stochastic technique to simulate noise mapping and to predict the noise exposure level in a workplace. A stochastic simulation framework and software, namely RW-eNMS, were developed to facilitate the random walk approach in noise mapping prediction. This framework considers the randomness and complexity of machinery operation and noise emission levels. Also, it assesses the impact of noise on the workers and the surrounding environment. For data validation, three case studies were conducted to check the accuracy of the prediction data and to determine the efficiency and effectiveness of this approach. The results showed high accuracy of prediction results together with a majority of absolute differences of less than 2 dBA; also, the predicted noise doses were mostly in the range of measurement. Therefore, the random walk approach was effective in dealing with environmental noises. It could predict strategic noise mapping to facilitate noise monitoring and noise control in the workplaces.

## Introduction

Noise pollution has become a global issue over the past half century because chronic noise exposure induces hearing loss and tinnitus in some individuals and affects their quality of life [1–3]. It interrupts daily activities and harms human health leading to such results as acoustic neuroma, annoyance, cognitive deficits, decreased physical well-being, stress, reduced performance, and compromised communication [4–7]. It also affects neighbouring wildlife by disrupting daily interactions, biodiversity, and ecological processes [8–12]. In general, noise mapping has been widely used for providing information for environmental noise impact assessment, increasing public awareness, identifying high-noise areas, and enabling noise control and monitoring [13–16]. The European Union (EU) issued Directive 2002/49/EC to focus on environmental noise assessment and management. It emphasises the utilisation of strategic noise mapping to cope with current environmental noise problems [17]. Practical guidelines were established by the European Commission Working Group to guide the development of production of strategic noise mapping [18]. Many recent studies have successfully developed strategic noise mapping in urban environments [15, 16, 19–24].

In current industrial practice, two-dimensional noise mapping is plotted for the workplace and used as information for preparing initial noise monitoring reports. Authorities use the noise map to identify high-noise area for conducting personal noise exposure measurements. However, the development of strategic noise mapping needs to be concerned with workplaces because current industrial practice is inaccurate and unreliable. Authorities lack concern about the utilisation of strategic noise mapping in working environments. Also, there are no standardised procedures or guidelines for developing strategic noise maps in the workplace. Undeniably, occupational noise exposure problems are increasing dramatically every year. Thousands of workers from different industries are exposed to noise levels above the threshold limits [25, 26]. Previous studies have revealed that severe hearing impairment is found among workers who are overexposed to excessive noise in the workplaces [27–29]. An environmental impact assessment prepared by Zolfagharian [30]; showed that the construction industry is the second highest source of environmental impact of noise pollution in Malaysia. The main reason for noise pollution in construction is high noise emission levels from heavy machinery and pneumatic tools [31, 32]. There are different stages in construction, and the excavation stage is the noisiest [33]. The presence of heavy machinery has increased the noise level significantly in construction areas. Many workers have been exposed to harmful levels of noise, which has caused negative effects to their hearing systems because of the ineffectiveness of noise abatement in construction [34–36]. Therefore, the development of noise mapping is very important because it could identify the current noise situation, provide useful information for future prediction, and assess the impact of noise in construction areas [13].

In particular, field measurement is used in construction activities by selecting several critical locations, measuring the noise levels, and comparing them to the permissible levels for further preventive actions. This method is used only with existing construction activities, but it is not possible to obtain actual noise levels during the planning process, before construction activity starts [37]. Two types of machines can be seen in the workplace: stationary machines and moving machines. It is difficult to construct noise mapping for construction processes because of variations in activities and random movements and duty cycles of earth-moving machines. Several models have been used to predict machinery operating performance and productivity, such as statistical analysis [38], artificial neural networks [39], and intelligent fuzzy models [40]. The locations and cycle times of earth-moving machines are always unpredictable. To study the random movements and cyclic times of earth-moving machines, an automatic spatiotemporal analysis was conducted using a global positioning system (GPS) to obtain real-time positions and machinery operating areas [41]. Although the moving paths of earth-moving machines are random and unpredictable, the working areas of earth-moving machines can be predicted during the planning process. The working areas of moving machines need to be identified because the operation and movements of machinery are the key contributors to fluctuations in noise levels. Haron [42] summarised the factors that affect the temporal distribution of noise levels in the workplace, including machinery sound power levels, duty cycles, locations of machines, and number of operating machines.

In fact, noise levels could be reduced by executing a strategic noise management during the planning process [43, 44]. This implies that noise prediction is the key managerial tool for predicting noise levels and monitoring machines for future activity. In recent years, many methods have been used to predict noise levels in the workplace, such as the discrete-event simulation method [37], artificial neural networks [45], regression analysis [46], the probabilistic approach [47], the simple prediction chart technique [48], and the stochastic Monte Carlo method [49]. These are the fundamental noise prediction methods that can be used as a managerial tool in the construction process. The stochastic approach can be reliably used to predict environmental noise by considering the randomness and complexity of the working environment [49]. Some studies have shown that consideration of dynamic construction activities is required in the noise prediction process, especially the random movements and duty cycles of machines. For example, the stochastic Monte Carlo approach considers the random position and sound power levels of machines in the prediction process. The randomness of machinery sound power levels is simulated according to the operating probabilities, including the probability of "Off", "Idle", and "Full" power [42]. Nevertheless, current methods consider noise prediction for a single receiving location only. A repetitive process is needed to predict the noise levels at other new receiving locations. Hence, further study is needed to predict a strategic noise map to indicate the spatial and temporal distribution of noise circumstances in working areas.

The present study introduced the use of the random walk approach for the prediction of strategic noise mapping and personal noise exposure in the workplace. Development of a new stochastic simulation framework and procedures to predict strategic noise mapping were discussed. Simulation algorithms and software interfaces were successfully programmed using the MATLAB. This approach considered the randomness of activities as well as the spatial and temporal distribution of noise levels. Interestingly, this approach was able to predict strategic noise mapping from moving machines and to simulate random movement of workers in working area for noise exposure prediction. Three case studies were conducted for data validation by comparing the prediction and measurement data. The simple prediction chart technique was used to compare the results of this approach. Most importantly, this paper revealed the accuracy, efficiency, and effectiveness of the proposed approach to enhancing current noise prediction methods.

### Strategic Noise Mapping

Strategic noise mapping is defined as noise maps generated by noise sources used for noise exposure assessment [17]. The EU directive stated that strategic noise mapping is used for public information and action plans for future noise mitigation. Mapping data can be obtained from measurements or prediction. However, the directive appears to be applicable in urban noise mapping development by involving general noise sources from transport infrastructure and industrial activity sites. The present study used noise emission levels from machinery to predict strategic noise maps in workplaces. Strategic noise map are very important in workplaces because they provide good visualisation of noise pollution in landscapes. The contour lines in the map represent the noise levels for each bounded area [50, 51]. Information can be obtained regarding patterns of noise distribution from various sources and the areas of noise pollution. The pattern of noise contour allows the description of noise sources, which is useful for future planning of noise mitigation [52]. Strategic placement of new machines could be selected based on noise mapping information, which could minimise noise levels when combining new sources and provide the best ergonomic conditions in the workplace [53]. This information solves facility location problem and also enhances safety system design. Data from noise mapping could be compared with permissible levels from regulatory standards and reveal the areas of noise-pollution, that are over permissible limits [13, 20]. For example, noise mapping data can be used for comparison with environmental noise limits from the Department of Environment (DOE). Also, assessment of community responses to environmental noise can be calculated by adopting the computing process from the DOE planning guidelines [54]. Moreover, noise mapping data can predict noise risk zones and classify risk circumstances in mapping areas [55]. It indicates sensitive spots by adopting noise risk evaluation criteria, as shown in Table 1, and discloses the percentage of risky areas [56].

**Table 1:** Noise Risk Zones [56].

Intensity of noise, dBA	Category of the zones
<66	Safe
66–71	Tolerable
71–76	Low risk
76–81	Moderate risk
81–86	High risk
>86	Extremely high risk

Using a computer to program the prediction framework could speed up the calculation process and plot a better visual contour map. The accuracy and precision of computational procedures are important. Asensio [57] pointed out that the size of grid refinement on a mapping area affects the accuracy of noise mapping data and the smoothness of contour lines. Although, an increase in grid resolution increases the accuracy and smoothness of contour lines, the computation process takes longer. Furthermore, field measurements for noise mapping are laborious and expensive, and disturb work progress. To address these problems, Nanthavanij [50] meticulously discussed a theoretical conversion of sound power to sound pressure level (SPL) and a computational analytical procedure for the development of noise mapping. The initial steps of noise mapping development are defining the *x* and *y* coordinates for the location of machines, the background noise, the sound power of machines and estimating the location of the combinations of noise levels. Then, the following computational steps are used to calculate the noise levels in a mapping area:

$$r_{ik}=\sqrt{{\left(x_{i}-x_{k}\right)}^{2}-{\left(y_{i}-y_{k}\right)}^{2}}$$ 1

$$I_{ik}=\frac{W_{k}}{2\pi r_{ik}^{2}}$$ 2

Eq (1) calculates the Euclidean distance *r*_*ik*_ from the machine location *k* to the specified location *i*. Then, Eq (2) calculates the machine noise intensity *I*_*ik*_, in W/m^2^, assuming the noise sources are above a hard surface and no power is absorbed by the surface [58], where *W*_*k*_ is the sound power (W) of the machine at location *k*.

$${\bar{I}}_{i}=I_{BG}+\sum_{k=1}^{m}\frac{W_{k}}{2\pi r_{ik}^{2}}$$ 3

The combination of machine noise intensity ${\bar{I}}_{i}$can be calculated by the summation of the noise intensity of all machines at a specified location *i* as shown in Eq (3), where *k* = 1, 2, 3,…, *m*. Meanwhile, the combination of machine noise intensity ${\bar{I}}_{i}$ needs to include the effect of background noise by adding the intensity of background noise *I*_*BG*_ into the Eq (3).

$$L_{p}{}_{\left(i\right)}=10\log\left(\frac{{\bar{I}}_{i}}{I_{0}}\right)$$ 4

The sound pressure level *L*_*p*(*i*)_, in dBA, at specified location *i* can be calculated from Eq (4) and *I*_*0*_ is the reference sound intensity, where *I*_*0*_ = 10^–12^ W/m^2^.

However, the analytical procedure disregards the effects of sound attenuation. Li [59] specified that the fundamental factors of sound attenuation should be included in the computations for noise prediction, such as geometric distribution, directivity and enclosure correction, barrier attenuation, air absorption, wind and temperature correction, and ground absorption. Obviously, those factors involve a lot of empirical data and lengthy computational algorithms, which makes it time-consuming and costly to construct a noise map. In the end, the present study made some assumptions about the development of strategic noise mapping. It assumed that the point sources are the areas 1 meter between the interval grid data and all noise sources above a hard surface. To speed up the prediction process, it adopted a simple approximation of barrier effects from BS5228-1:2009 to calculate noise reflection and reduction [60]. This was because barrier effects are major factors affecting propagation of noise. In other words, the present study ignored the fundamental factors in the simulation process, but considered barrier effects by applying a simple approximation to determine barrier attenuation.

### Stochastic Modelling

In general, random variables are obtained by a sequential process by applying probability theory with a given time frame, defined as stochastic modelling [61]. The mathematical models can be divided into two models, stochastic and deterministic, in calculating the physical properties of noise [62]. Comparison of stochastic and deterministic models showed that the stochastic model had better performance and smaller mean absolute error, and reduced the overestimated error in output [63]. The stochastic model has cost advantages and provides a more realistic scenario compared to the deterministic model [64]. It could also reveal the uncertainty of a problem through a statistical analysis in stochastic modelling [65]. A previous study showed that the incorporation of the stochastic approach in noise modelling successfully determined free turbulent flows of sound waves [66]. Indeed, stochastic modelling effectively predicts environmental noise levels and is applicable during the planning process. By dealing with the complexity of dynamic construction activities, the stochastic model predicts the spatial and temporal distribution of noise levels from concurrent and nonconcurrent operations of multiple machines [67]. The outcomes from the simulation process can predict the equivalent continuous noise level (LAeq) with standard deviation for a receiving location. The probability distribution function (PDF) and the cumulative distribution function (CDF) can be produced by incorporating statistical analysis. A CDF chart can determine noise indices (L10, L50, and L90), which are important parameters for environmental noise assessment [47]. Haron [48] developed a simple prediction charts technique based on stochastic modelling. The noise level at the receiving location can be easily predicted by estimating the site aspect ratio, obtaining the sound power of machinery and using a simple equation. The parameters of this equation can be referred to the prediction charts, such as the mean noise level and standard deviation.

The random walk approach is a well-known stochastic process applicable in many fields, for example, physics, chemistry, biology, finance, and economics [68–76]. It is also known as the basic theory of diffusion and Brownian motion. The principles and formulations of this approach were discussed in detail by Spitzer [77]. The basic concept describes a walker moving in a succession of random steps with discrete times in a two*-*dimensional Euclidean plane. Every new location depends on the previous location, but is independent of the previous moving direction. The following equations describe the concept of the random walk approach [78]:

$$R_{n}=R_{0}+\sum_{k=1}^{n}r_{k}$$ 5

In Eq (5), *R*_0_ is the initial location of a walker at time *t*_0_. After walking through a sequential time *t*_1_, *t*_*2*_, *t*_3_,…, *t*_*n*_, the walker has passed through successive displacements *r*_1_, *r*_2_, *r*_3_,…, *r*_n_. The *R*_*n*_ is the final location of the walker.

$$p_{k}(r_{k})dr_{k}=Pr\left\{r\leq r_{k}\leq r+dr\right\}$$ 6

In Eq (6), {*r*_*k*_} is the independent variable and is determined by considering the probability function at the interval of (*r*, *r* + *dr*).

$$P(x,y)\partial x\partial y=\mathrm{Pr}\left\{x\leq x(\theta_{j})\leq x+\partial x,y\leq y(\theta_{j})\leq y+\partial y\right\}$$ 7

$$x(\theta_{1}...\theta_{n})=\sum_{j=1}^{n}a_{j}\cos\theta_{j}$$ 8

$$y(\theta_{1}...\theta_{n})=\sum_{j=1}^{n}a_{j}\sin\theta_{j}$$ 9

Pearson's random walk model describes the movement of a walker with random directions in the *x* and *y* axes. By considering the PDF in Eq (7), the successive lengths *a*_1_, *a*_2_, *a*_3_,… *a*_*n*_ can be determined at the sequential angles *θ*_1_, *θ*_2_, *θ*_3_,… *θ*_n_. Eq (8) and (9) calculate a new location of a walker in *x* and *y* coordination. The two directions are considered in the development of a stochastic simulation framework, because current industrial practice uses two-dimensional noise mapping to obtain information for documentation and dissemination.

### Stochastic Simulation Framework

**Fig 1:**
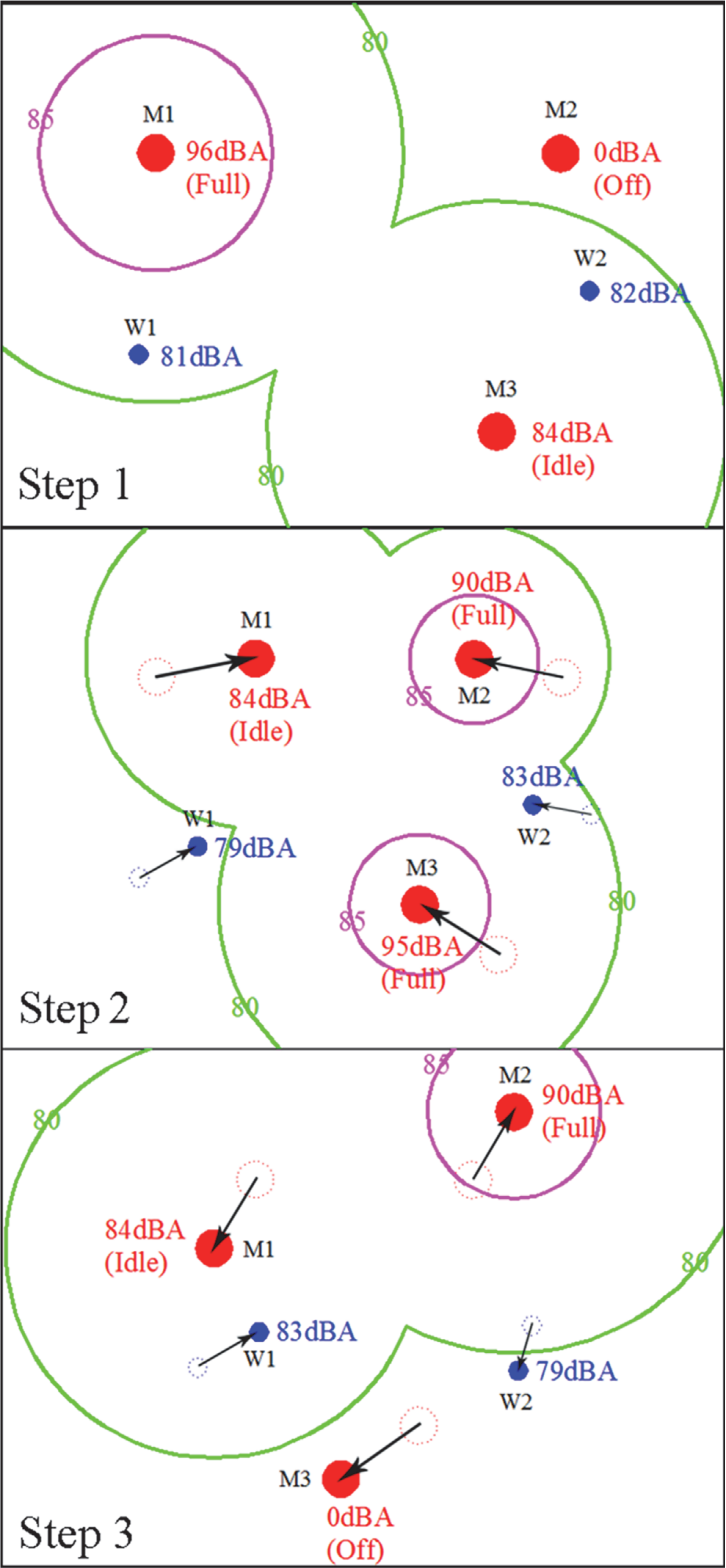
Concept of Random Walk Simulation.

In the present study, a new concept was developed by using the random walk approach with stochastic modelling to simulate the noise circumstances in a dynamic working environment. The random walk approach was applied to simulate the random movement of machines and workers in the mapping area. Fig 1 shows the concept of the simulation process using the random walk approach. The red and the blue circles represent the moving machines and workers, respectively. In step 1, the initial location of machines and workers before they moved into step 2 is indicated. The machinery sound power levels were predicted by implementing the probability approach to model the randomness of duty cycles during the working period. The moving machines M1, M2, and M3 showed their operating status and different sound power levels in step 1. A noise contour was plotted on the mapping area to indicate the noise levels emitted by the operating machines. The noise exposure level of workers W1 and W2 could be obtained from the noise map and recorded for further implementation. Then steps 2 and 3 illustrated that machines and workers were moving in random directions. Every new position depended on the previous position to reflect the dynamic circumstances in the workplace. The noise contours were also changed in steps 2 and 3 to indicate the random variation of machine cycle times. Similarly, the noise exposure levels varied in every new step, which indicated that the workers were moving randomly and were exposed to different noise levels in the working area. After the simulation process, computation of LAeq was carried out for every receiving location, and the results were used to plot strategic noise mapping. The noise dosages could be predicted by using the noise exposure levels of workers in the simulation process.

#### Descriptions

**Fig 2:**
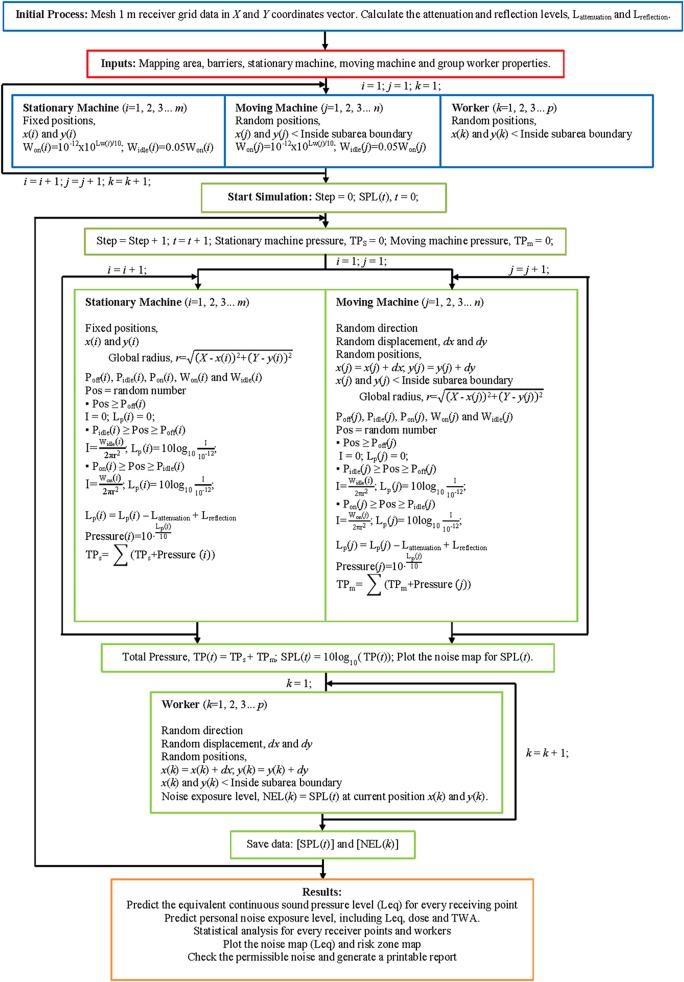
Framework of Random Walk Simulation in Noise Mapping Prediction.

The stochastic simulation framework was established by using the random walk approach to predict a strategic noise map, as shown in Fig 2. It was a simulation process that improved the stochastic modelling by eliminating repetitive processes in predicting numerous receiving locations. It could even plot a two-dimensional noise contour. In this section, the stochastic simulation framework is described by using the simple random walk approach in noise mapping prediction. Some inputs needed to be defined before starting the simulation process. The important inputs used in the simulation process were as follows:

- Mapping area: site layout, width and depth of mapping area, background noise, and working periods
- Barriers: location, width, and depth of barriers
- Stationary machines: location of noise sources, noise emission levels (Lw), and probabilities of duty cycles
- Moving machines: location, width and depth of subarea, number of moving machines, noise emission levels (Lw), and probabilities of duty cycles
- Group workers: location, width, and depth of subarea

In the initial process, the dimensions of the mapping area were used to mesh the grid points in *X* and *Y* coordinates vectors and a 1-meter interval between two grid points was assumed. For example, a 100 m × 70 m (global width × global depth) mapping area with a 1-meter interval between grid points would yield a vector matrix with a size of 101 × 71. The grid points in *X* and *Y* were important for further application, where they were used to predict the noise levels for every grid point in the mapping area before plotting the noise contour. On the basis of the BS5228-1:2009 [59], it was stated that a simple working approximation could be used to predict the noise attenuation and reflection levels due to barriers between the noise sources and the receiving points. Table 2 shows the assumption of noise attenuation and reflection levels due to barriers or any topographic features. Then a looping process arranged the fixed position of stationary machines and predicts the initial random position for moving machines and workers inside the boundaries of subareas. Looping continued until all machines and workers were taken into account. In Fig 2, *m*, *n*, and *p* represent the total number of stationary machines, moving machines, and workers in the prediction, respectively.

**Table 2:** Noise Attenuation and Reflection Due to Barriers from BS5228-1:2009 [60].

Condition	Approximation
Source is visible to the receiver	Attenuation −5 dB
Source is hidden from the receiver	Attenuation −10 dB
1 m from the facade of a building	Reflection +3 dB

**Fig 3:**
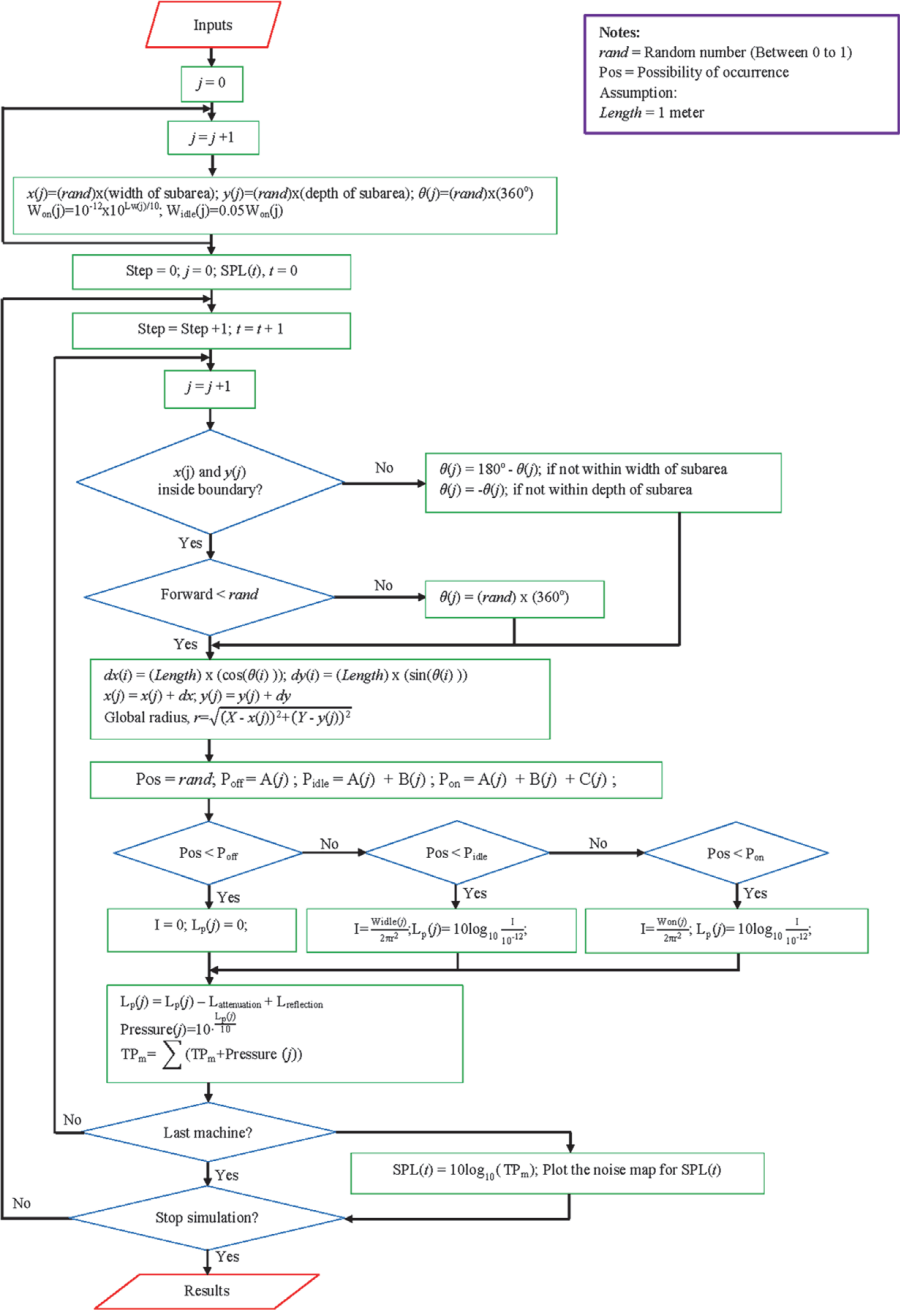
Programming Algorithm for Moving Machines.

After completion of the initial process, the random walk simulation started the walk step from 0 to 1. A looping process on the stationary machines and moving machines was carried out to predict the SPLs on the whole mapping area. The difference in this process between stationary machines and moving machines was their positions, where stationary machines were always in a fixed position and the moving machines were always changing position randomly. The random walk approach was used to simulate new positions for moving machines with random directions and random displacements. The new random positions for every moving machine needed to be limited to their subareas as defined from the input. Fig 3 shows the programming algorithm used in predicting the noise map for moving machines. The global radius was the distance between the receiving grid data and the noise source position. Likewise, the randomness of acoustic power in predicting machine emission levels was based on the probability of machine duty cycles, such as "Off" (A), "Idle" (B), and "Full" power (C) cycles. Assuming hemispherical radiation from noise sources over the hard surface, the global radius and acoustic power of noise source were used to calculate the sound intensity from a source to all receiving points and then the SPL (Lp) was computed by including attenuation and reflection levels (L_attenuation_ and L_reflection_) due to barriers. The final step in this looping process was the conversion of the SPL into pressure and the accumulation of stationary and moving machine pressures. Looping continued until all machines were taken into the process.

Furthermore, the next process was the summation of stationary and moving machine pressures to predict the overall SPL and plot a noise map for the current simulation step where the noise contour was plotted by using the linear interpolation method between the nodes. Meanwhile, the random walk approach could be applied to the prediction of personal noise exposure levels. Another looping process was carried out to predict a new position for every worker in the simulation. The noise levels were automatically obtained from the predicted SPL in that particular position and noise data was stored for predicting noise exposure levels at the end of the simulation process. Also, the noise mapping data or SPL in the current simulation step was saved. It was an iterative process, and the simulation step was increased by adding one to every new step.

The last part of this framework was to calculate the LAeq for every receiving point. Thus, the LAeq was used to predict the noise map and represented the final result for this simulation process. Also, the personal noise exposure levels, including the noise doses and the time-weighted average (TWA) sound levels, could be predicted based on the working periods as defined from the input. A statistical analysis was applied in this part to compute the standard deviation, the maximum and minimum noise levels, and other noise parameters for each receiving points. The SPLs, histogram, PDF, and CDF were analysed and plotted in more detail. The PDF revealed the density of a continuous noise level distribution by considering machine duty cycles and random positions. Similarly, the CDF showed the probability distribution in multivariate random noise levels from the simulation. On the basis of the CDF, the L50 (noise level exceeded for 50% of the time), L10 (noise level exceeded for 10% of the time), and L90 (noise level exceeded for 90% of the time) could be predicted. Those are important parameters for noise monitoring. In addition, the environmental standard from DOE were incorporated to check the permissible noise level in different receiving land use categories and to assess community annoyance responses [54]. Lastly, noise risk zones could be predicted according to the different levels of noise intensity from Table 1. Extremely high risk areas in the workplace were indicated and that information could be used for further noise monitoring and abatement.

#### Strategic Noise Mapping Prediction Simulation Software (RW-eNMS)

**Fig 4:**
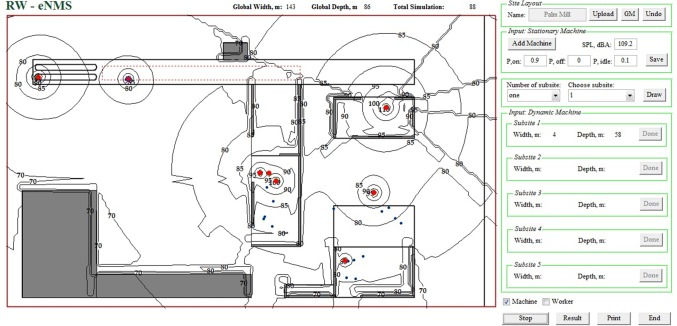
Random Walk Simulation Software (RW-eNMS).

To increase prediction performance, MATLAB [78], powerful programming software efficient in calculating matrix data, was used to program the simulation algorithms. New strategic noise mapping prediction simulation software, namely RW-eNMS, was developed by using the interactive GUIDE tools. The graphical user interface (GUI) is presented in Fig 4. This simulation software is facilitated in the stochastic simulation framework to predict a strategic noise mapping. It allows users to define noise sources, workers, and barriers in the workplace. During the simulation process, it plotted a noise map to reflect the noise circumstances for every new simulation step. Users could increase their understanding based on the variation of noise propagation and know the impact of noise on the surrounding environments. After the simulation process, it could predict noise mapping for LAeq and personal noise exposure level. The noise contours were filled with different colours to represent different ranges of noise level and to enhance the visualisation of noise mapping for users. Users could obtain the noise data from every receiving location by clicking on the mapping area in GUI. Meanwhile, this software was incorporated into the environmental regulation, so users could easily check whether the noise level was under or over the permissible level. It also mapped the noise risk zones to indicate the hazardous areas and determines the percentage of noise pollution in the working areas. In addition, this software could generate a printable noise map for documentation and further implementation.

## Data Analysis and Validation

**Fig 5:**
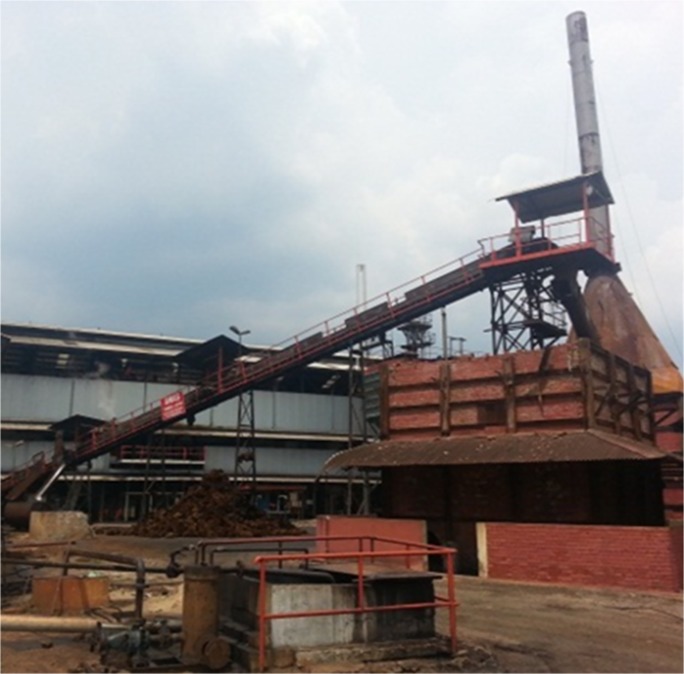
Mapping Area of Oil Palm Mill.

**Fig 6:**
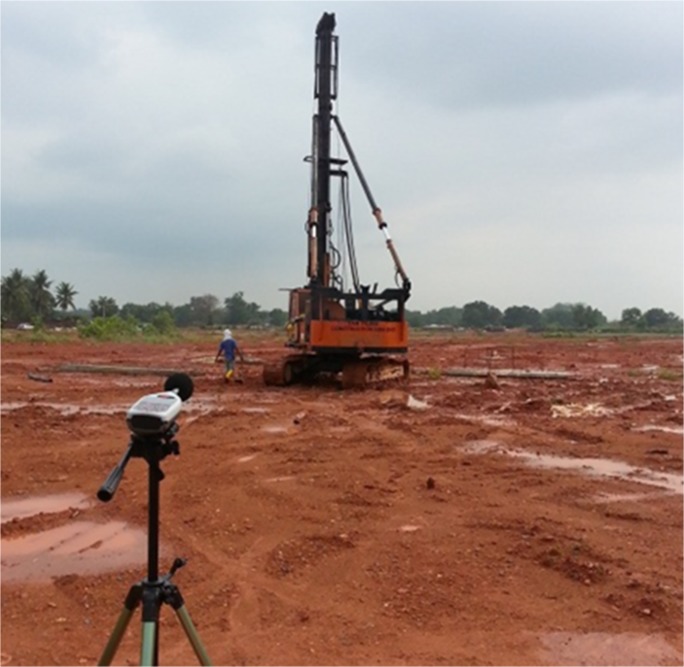
Mapping Area of Piling Activity.

**Fig 7:**
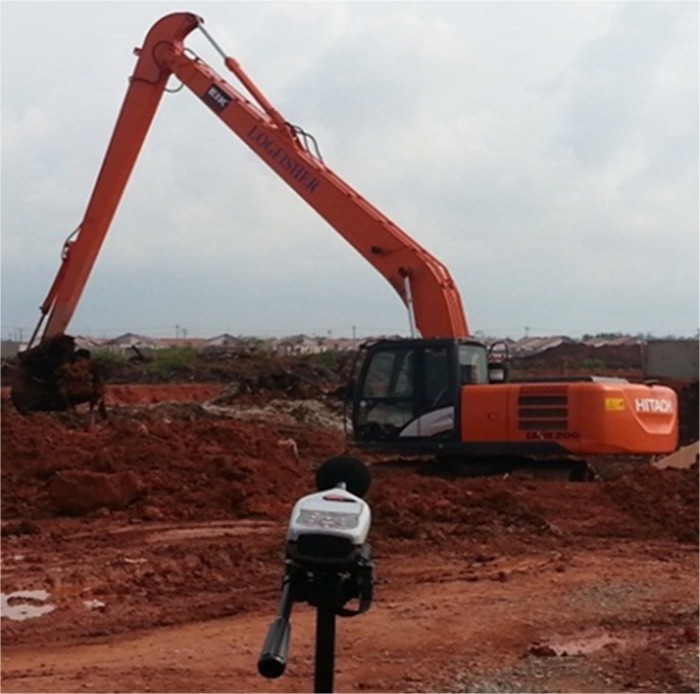
Mapping Area of Drainage Excavation.

This section discusses the results of the case studies and compares the measurement and prediction data. It is of crucial importance to validate the accuracy of prediction data and unveil the efficiency and effectiveness of the random walk approach in noise mapping prediction. Three case studies were carried out at an oil palm mill and two construction sites in southern Malaysia. Figs 5, 6 and 7 show the mapping areas to be measured at different workplaces during the working periods. The measurements were derived by using a sound level meter (Pulsar Model 33 Type 1), a sound level meter (3M SoundPro SE/DL Type 2), and a dosimeter (3M the Edge). These devices conform to the recommendations of the International Electrotechnical Commission (IEC), such as IEC 61672-1-2002 [80]. The devices were calibrated at the start and end of measurement. The noise data was recorded in an A-weighting frequency network at a height of 1.5 meter for every measurement points during the working period. Determination of sound power levels (LWA) for stationary and earth-moving machines was based on the British Standards BS EN ISO 3744:2010 [81] and BS ISO 6393:2008 [82].

### Case Study 1: Oil Palm Mill

**Fig 8:**
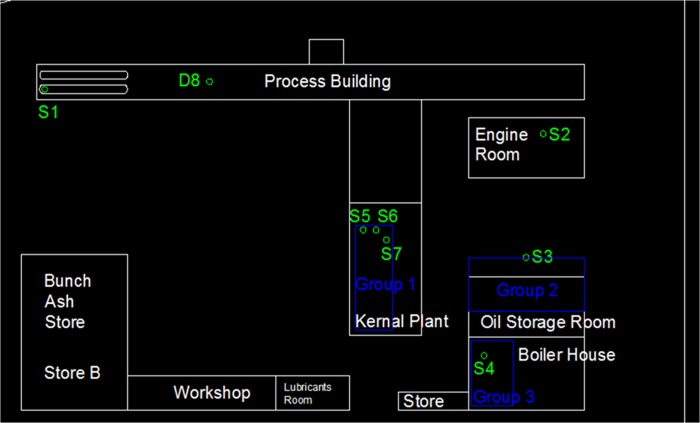
Oil Palm Mill Layout.

**Fig 9:**
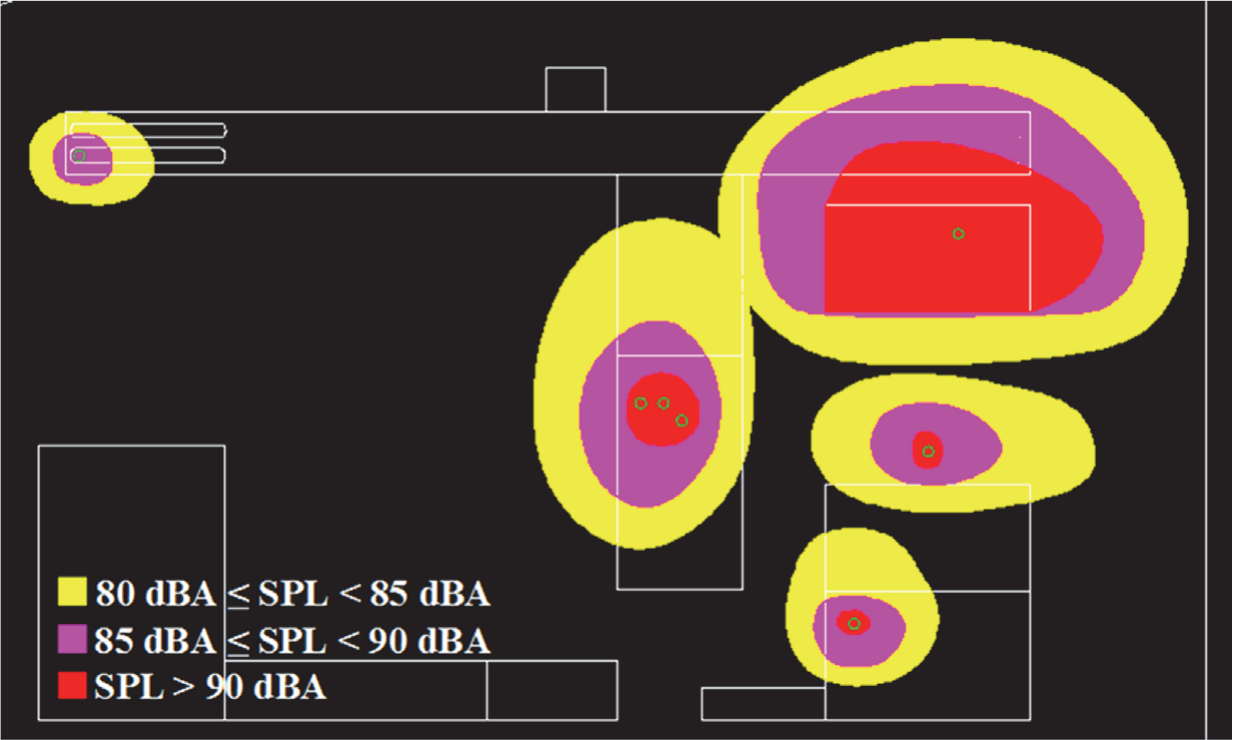
Oil Palm Mill Area Noise Map.

An oil palm mill was selected for measurement, and the objectives of this case study were to obtain noise data for validation, plot an area noise map, and measure personal noise exposure levels from different working areas. Fig 8 shows the layout of the palm mill and the positions of machines. The dimensions of the mapping area were 143 m × 86 m, and the background noise was approximately 52.2 dBA. First, machine emission levels needed to be obtained, which is significant input data for noise mapping prediction. Measurement was conducted on operating machines only, and nonoperating machines were not taken into account. In other words, the results represented the noise circumstances of the workplace during the measurement period. Results could change for any additional operating noise source. A total of seven stationary machines and one earth-moving machine were operating during the measurement period, and their sound power level and duty cycles are shown in Table 3. An area noise map for the workplace was plotted by applying the current industrial practice in Malaysia. This was due to nonstandard procedures or methods in area noise mapping for the workplace. Furthermore, an equivalent continuous noise level (LAeq) was measured and recorded at 42 measurement points, with a duration of 15 seconds each. All measurement points were located surrounding the stationary machines to plot an area noise map. The noise intervals to be measured were approximately 80, 85, and 90 dBA. There was an earth-moving machine in this case study only, and obtaining its fixed noise interval was impossible because of the continuous movement of the machine during the working period. The area noise map was plotted by using the AutoCAD software, as shown in Fig 9.

**Table 3:** Machinery Sound Power Levels and Duty Cycles.

CS	Machine	C	L_WA_, dBA	P_on_	P_off_	P_idle_	L
1	Exhaust pipe	S	103.9	0.9	0.0	0.1	S1
1	Generator	S	119.7	0.9	0.0	0.1	S2
1	Oil purifier	S	105.0	0.9	0.0	0.1	S3
1	Conveyor 1	S	105.0	0.9	0.0	0.1	S4
1	Conveyor 2	S	109.2	0.9	0.0	0.1	S5
1	Silo fan 1	S	106.5	0.5	0.5	0.0	S6
1	Silo fan 2	S	106.5	0.5	0.5	0.0	S7
1	Narrow gauges	EM	96.8	0.7	0.0	0.3	D1
2	Piling machine	EM	111.1	0.3	0.1	0.6	D2
3	Excavator	EM	98.8	0.7	0.0	0.3	D3

*Note*: CS, case study; C, characteristic; L, label; S, stationary machine; EM, earth-moving machine.

**Fig 10:**
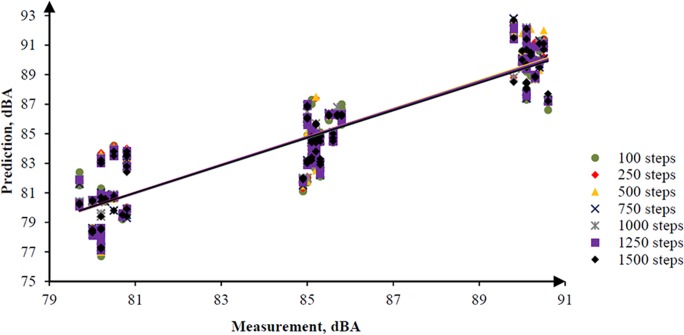
Correlation between the Prediction and the Measurement Data in Case Study 1.

**Fig 11:**
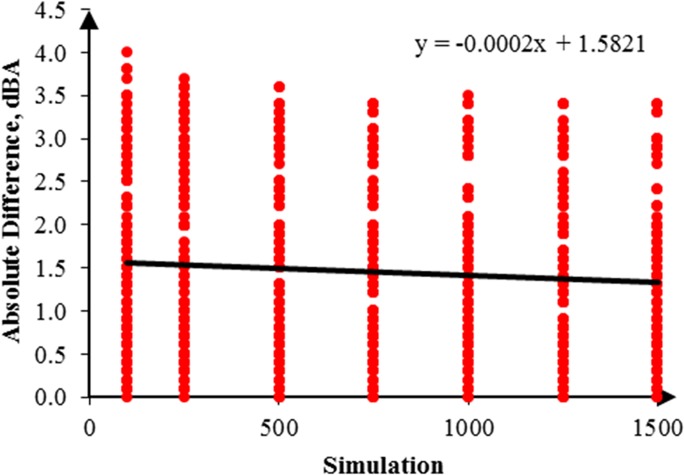
Absolute Difference for 1,500 Steps in Case Study 1.

In addition, the RW-eNMS was used to simulate a noise map. On the basis of the predicted noise map, it could obtain prediction data from the map to compare with the measurement data. The simulation of noise mapping in the oil palm mill was repeated with different simulation steps, for instance, 100, 250, 500, 750, 1,000, 1,250, and 1,500 steps. Also, every simulation step was repeated three times to obtain more prediction data with similar simulation steps for validation. The reason simulation was stopped at 1,500 steps because the simulation result had reached the steady absolute difference value. A total of 882 prediction data points were predicted in this case study. Fig 10 shows a graph that was plotted to determine the goodness of fit of the prediction data compared with the measurement data. Similar graph was also used to determine the accuracy of the random walk approach in noise prediction as well as the influence of increments of simulation steps. It was found that the regression line was highly fit to the measurement data, but the squared correlation coefficient increased slightly with the increment of simulation steps, from 0.7818 (100 steps) to 0.8339 (1,500 steps), as shown in Table 4. Obviously, the majority of the machines in this case study were in fixed positions, and the variation of noise levels was based on duty cycles only. Likewise, the absolute difference between the prediction and the measurement results is plotted in Fig 11. A linear line with positive slope was found. It showed that the increments of simulation steps increased the accuracy of the data.

**Table 4:** Squared Correlation Coefficient.

Simulation steps	Squared correlation coefficient, *R* ^2^		
	Case study 1	Case study 2	Case study 3
100	0.7818	0.0373	0.5246
250	0.7930	0.0683	0.5423
500	0.7976	0.4513	0.7382
750	0.8045	0.4932	0.7435
1,000	0.8137	0.5156	0.7742
1,250	0.8190	0.5667	0.8099
1,500	0.8339	0.7375	0.9155

During the working day, 46 workers were exposed to noise in this oil palm mill. Hearing protection was provided to all workers, but it was observed that they did not wear it regularly. Personal noise exposure measurement was carried out in three critical subareas, as shown in the palm mill layout, namely, Group 1, Group 2, and Group 3. Most of the working activities were implemented in these subareas. Further, the dosimeter was used to measure the LAeq and noise dose percentage. The measurement was repeated four times for every subarea, and the duration of measurement was 30 minutes. Theoretically, the random walk approach assumes that workers are randomly moving and working in a subarea only, without considering that the worker moves outside the boundaries. It needed to be ensured that the measurements were conducted inside the boundary of the subarea with random movement. Hence, the average results of 1,500 simulation steps were compared with the average measurement results, as shown in Table 5. Both results considered that the workers were continuously exposed to noise in the three subareas within 30 minutes. The dosage differences were −0.2%, 1%, and 0.2%, where all differences were small and almost the same as the actual measurement results.

**Table 5:** Personal Noise Exposure of Measurement and Prediction for 1,500 Steps.

Group	Measurement		Prediction	
	LAeq, dBA	Dose, %	LAeq, dBA	Dose, %
1	88.4	4.4	89.2	4.2
2	85.9	2.4	85.1	2.5
3	88.5	4.4	92.0	4.6

**Fig 12:**
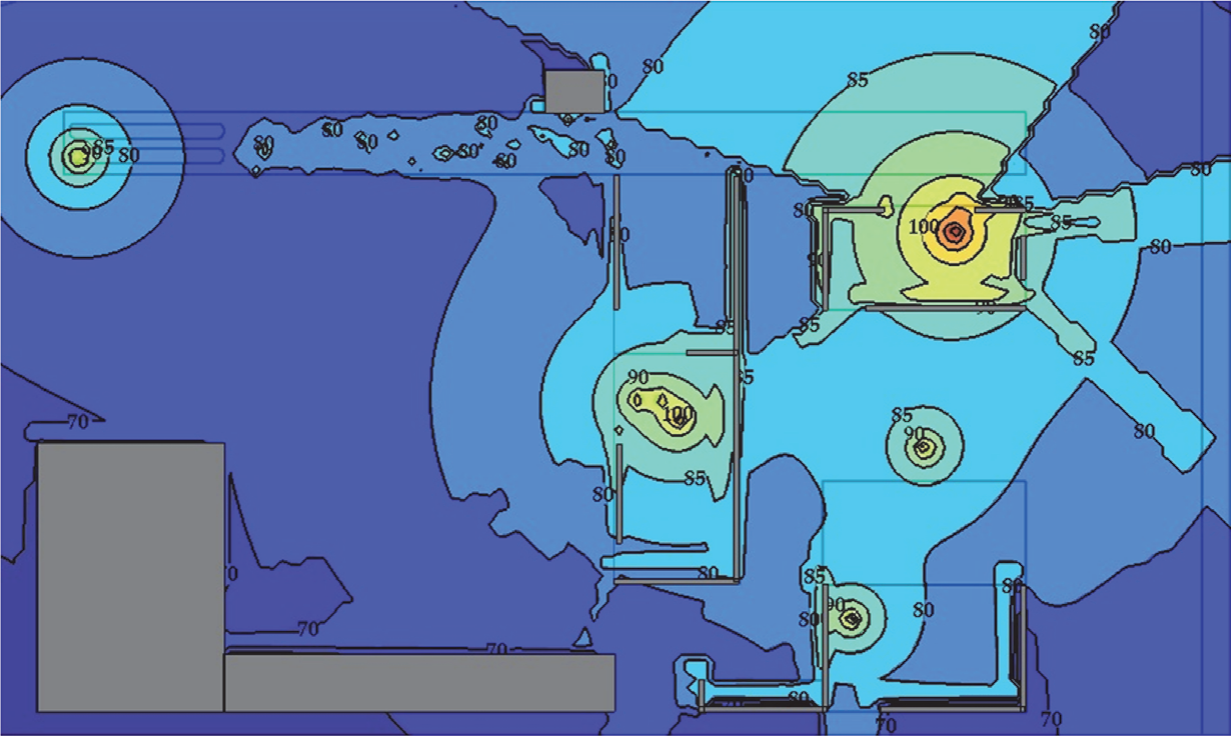
Strategic Noise Mapping with 1,500 Simulation Steps: Case Study 1.

**Fig 13:**
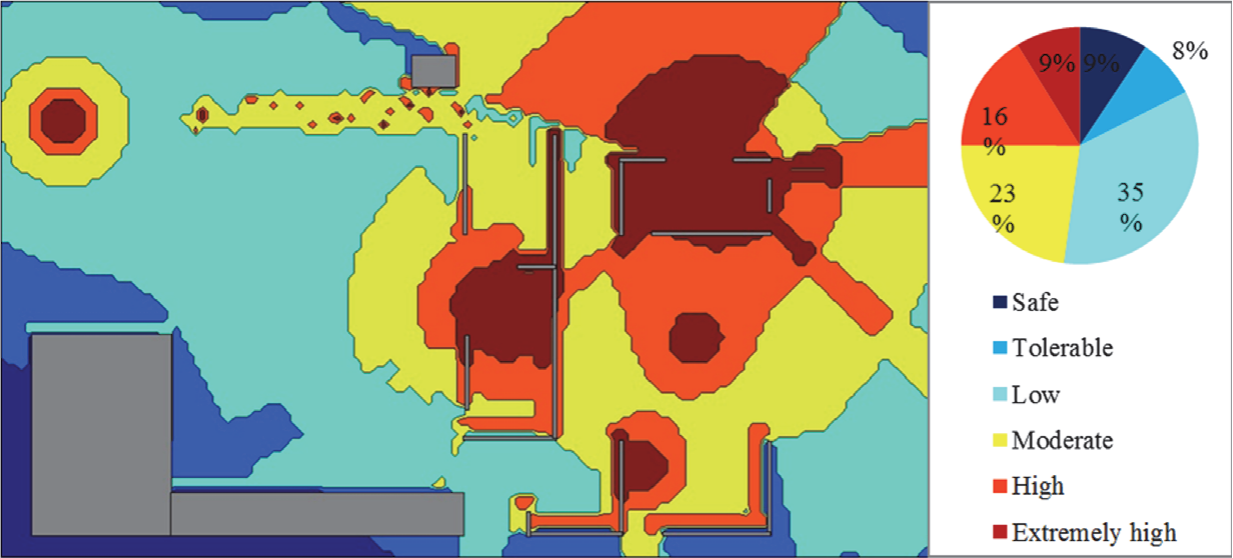
Noise Risk Zones with 1,500 Simulation Steps: Case Study 1.

Fig 12 shows a strategic noise map that was predicted by simulating 1,500 steps and by assuming an eight-hour working period. Contour lines with a 5 dBA interval were plotted, and intervals were filled in with various colours. On the basis of the strategic noise map, it could simply differentiate high-noise areas, for instance, the engine room, the kernel plant, the oil storage room, and the boiler house. In addition, a noise risk zone was plotted, as shown in Fig 13. It showed that the area was 9% extremely high risk and 17% high risk. Groups 1, 2, and 3 were working in these risky areas. The prediction result implies that workers are at risk of chronic noise exposure in these areas. Meanwhile, the prediction showed that Groups 1 and 3 were overexposed to noise. The average dosage percentages were 60.1% and 65.3%, respectively. The dosage of Group 2 was lower than 50%, but the average TWA was 83.3 dBA, which was close to the 85 dBA limit.

### Case Studies 2 and 3: Construction Sites

**Fig 14:**
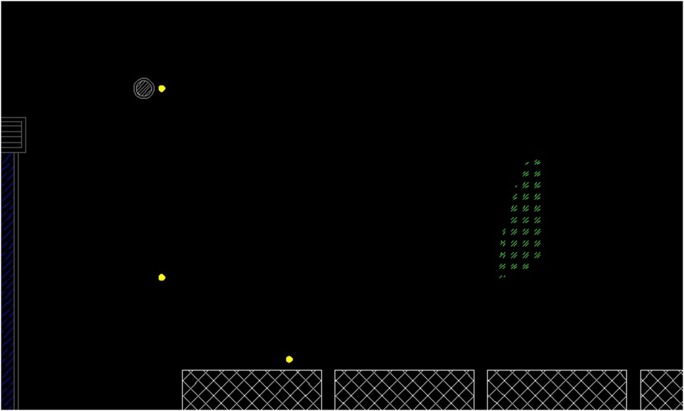
Piling Activity.

**Fig 15:**
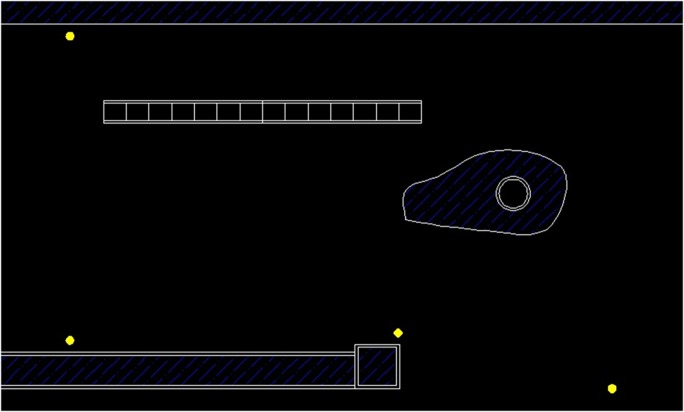
Drainage Excavation Activity.

The previous case study discussed the implementation of the random walk approach in the prediction of area noise map with a majority of stationary machines and personal noise exposure in a workplace. In this section, this approach is applied to predicting a noise map for earth-moving machines. In fact, random movement and the duration of work for earth-moving machines are unpredictable; so, area noise mapping methods cannot be used to obtain data for validation. Therefore, the measurements were carried out by assuming a boundary for the mapping area where the earth-moving machines were operating only inside the boundary. In other words, the measurement locations had to be around the activity, considering that no other external noise sources affected the final results. Several measurement points were selected to measure the LAeq with a duration of 15 minutes. There was a limited period for measurement because construction activity varies continuously. A short designated duration for measurement can be applied to activities with limited working period [54]. The background noise for both case studies was approximately 48.4 dBA. In Case Study 2, the measurements were implemented at a piling activity for the construction of a bungalow foundation. An earth-moving piling machine was operating during the measurement, and details are described in Table 3. The dimensions of mapping area were 100 m × 60 m, and three measurement locations were selected. Case Study 3 was an excavation activity to construct a new drainage system, and an excavator was operating during the measurements. Four measurement points were selected with the mapping area dimensions of 60 m × 36 m. Figs 14 and 15 show the site layouts of both studies, and the ‘yellow dots’ represent the locations of measurements.

**Fig 16:**
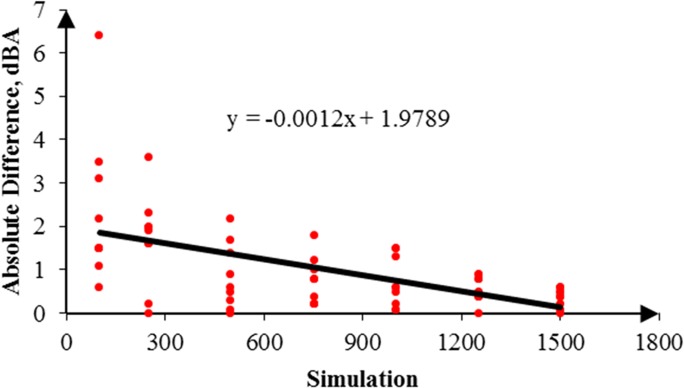
Correlation between the Prediction and the Measurement Data in Case Study 2.

**Fig 17:**
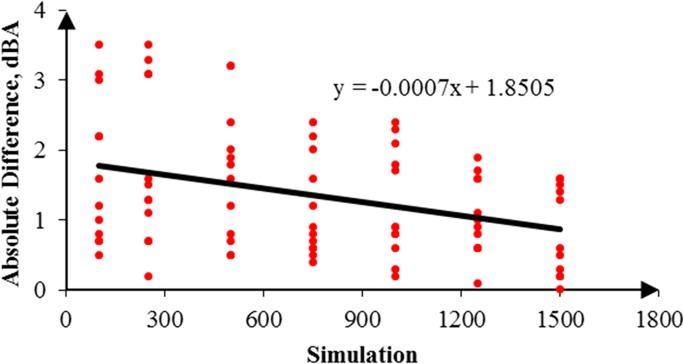
Correlation between the Prediction and the Measurement Data in Case Study 3.

**Fig 18:**
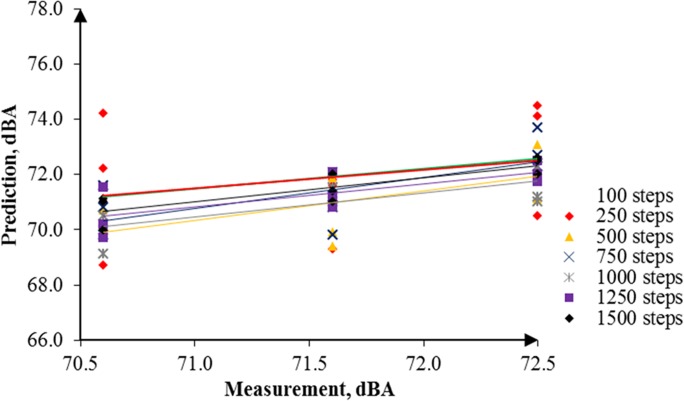
Absolute Difference between the Prediction and the Measurement Data in Case Study 2.

**Fig 19:**
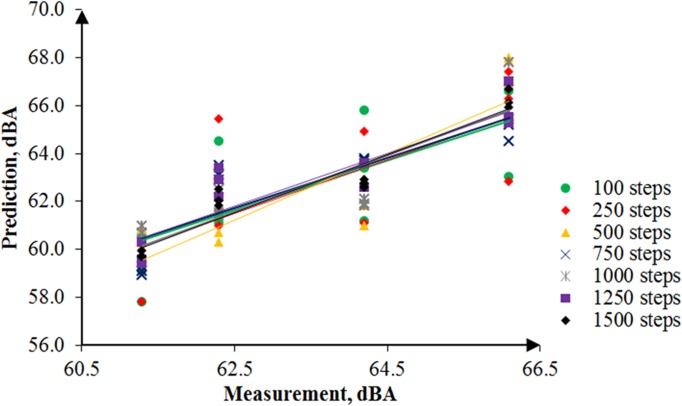
Absolute Difference between the Prediction and the Measurement Data in Case Study 3.

**Fig 20:**
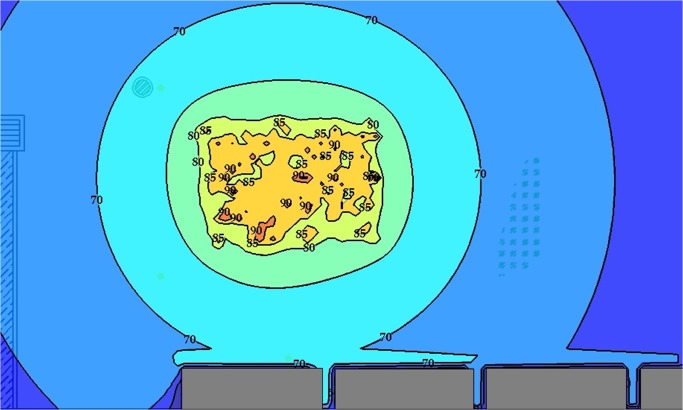
Strategic Noise Mapping with 1,500 Simulation Steps: Case Study 2.

**Fig 21:**
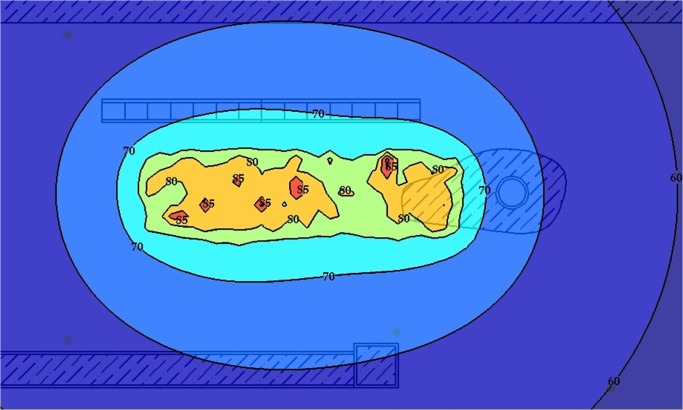
Strategic Noise Mapping with 1,500 Simulation Steps: Case Study 3.

**Fig 22:**
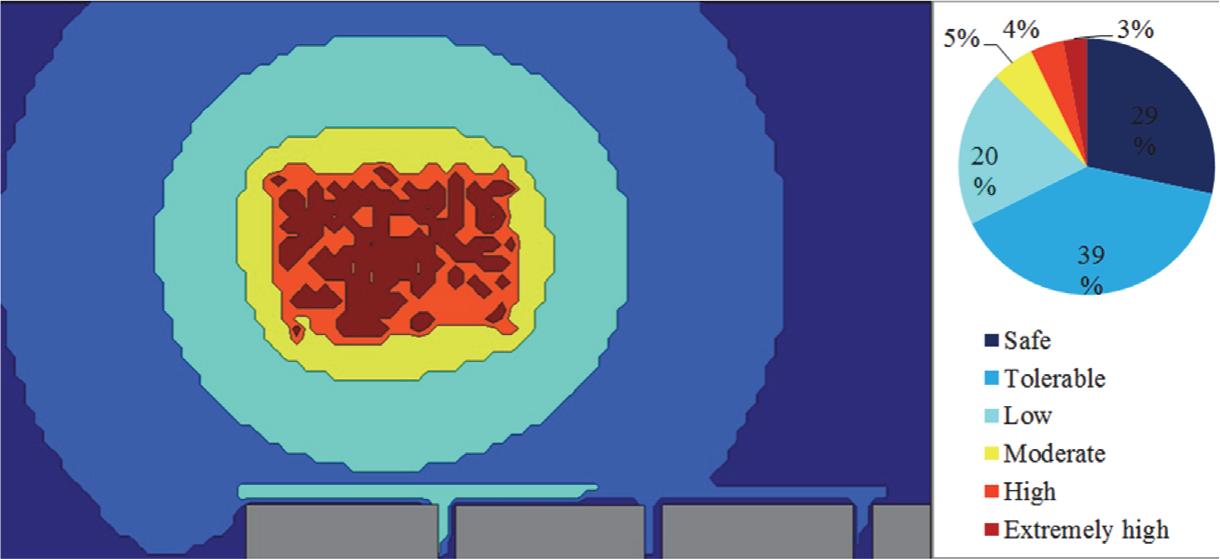
Noise Risk Zones with 1,500 Simulation Steps: Case Study 2.

**Fig 23:**
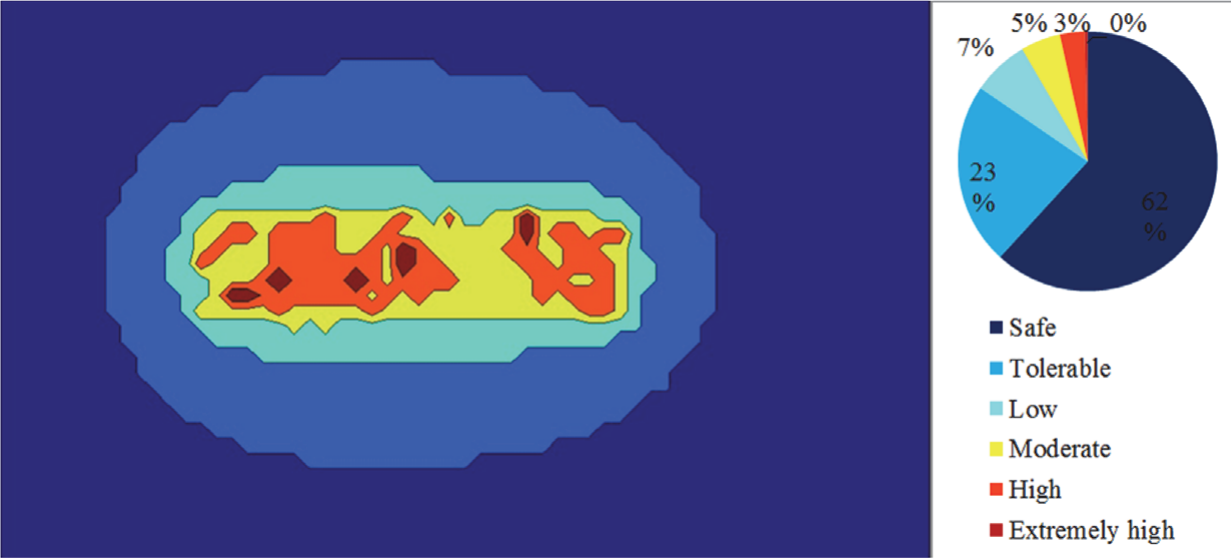
Noise Risk Zones with 1,500 Simulation Steps: Case Study 3.

By the same token, several noise maps with different simulation steps were simulated by using the RW-eNMS. The earth-moving machine was randomly moving under the subarea boundary constraints. After the simulation process was finished, the LAeq at the yellow dot locations were recorded, and were used to validate the measurement data at the same locations. The correlation and absolute difference graphs for both case studies were plotted for Figs 16, 17, 18 and 19. Both simulations were stopped at 1,500 steps, when the absolute difference had reached a steady observed value. On the basis of both correlation graphs, the squared correlation coefficients increased noticeably, from 0.0373 (100 steps) to 0.7375 (1,500 steps) in Case Study 2, as well as from 0.5246 (100 steps) to 0.9155 (1,500 steps) in Case Study 3. A negative slope in the absolute difference graph of both studies was found, which means the increase in simulation steps was needed to increase the accuracy of noise prediction for earth-moving machines. The regression lines were highly fit to the measurement results. The area noise maps and noise risk zones were predicted by implementing 1,500 simulation steps as shown in Figs 20, 21, 22 and 23. Both results revealed that the workers who were working closer to the machinery might have been at higher risk for hearing problems.

### Stochastic Simulation Framework vs. Simple Prediction Chart Method

In this section, the accuracy of the stochastic simulation framework compared with the prediction results of the simple prediction charts technique is discussed [48]. The principle of the simple prediction chart technique was discussed in the previous section. This technique was used to predict the noise levels at every measurement points in Case Studies 1, 2, and 3. The percentages of absolute difference were calculated and used to compare with the results of the stochastic simulation framework. Table 6 shows the total simulation results of 1,500 steps in Case Studies 1, 2 and 3. Case Study 1 showed that a difference of approximately 80% was ≤2 dBA. In detail, an absolute difference of approximately 48% was ≤1 dBA. However, the simple prediction chart technique predicted that an absolute difference of 52% was ≤2 dBA and an absolute difference of 31% was ≤1 dBA for Case Study 1. In Case Study 2, the maximum absolute difference was 0.6 dBA. Also, the largest absolute difference in case study 3 was 1.6 dBA. Both case studies obtained an absolute difference of less than 2 dBA. The simple prediction chart technique performed well in Case Study 2 with an absolute difference of 100% being ≤2 dBA. Still, it predicted an absolute difference of 100% was >3 dBA in Case Study 3 as shown in Table 6. Obviously, the stochastic simulation framework had performed better compared to the simple prediction chart technique. This framework eliminated the laborious process of calculating the noise levels for each receiving location, and provided more consistent and reliable results.

**Table 6:** Comparison of the Prediction Results between Stochastic Simulation Framework and Simple Prediction Chart Technique.

Absolute Difference, dBA	Case Study 1		Case Study 2		Case Study 3	
	SSF, %	SPC, %	SSF, %	SPC, %	SSF, %	SPC, %
≤ 0.5	25	21	78	33	42	-
≤ 1.0	23	10	22	-	8	-
≤ 1.5	13	19	-	-	25	-
≤ 2.0	19	2	-	33	25	-
≤ 2.5	5	14	-	33	-	-
≤ 3.0	12	14	-	-	-	-
> 3.0	3	19	-	-	-	100

*Note*: SSF, stochastic simulation framework; SPC, simple prediction chart method;

## Discussion

Applying the concept of the random walk approach allowed simulation of random movement of machinery and workers to reflect the actual circumstances. This approach considered the randomness of machine emission level and duty cycles, such as "Off", "Idle", and "Full" power operating cycles. Also, the complex interaction of daily working activities was taken into consideration, including concurrent and nonconcurrent activities. Interestingly, this approach was able to predict a noise map for moving machines and occupational noise exposure levels. The results of the three case studies showed that the proposed approach was effective in predicting occupational noise levels with high accuracy and yielded small absolute difference compared with the noise levels from actual measurements. The majority of absolute differences were less than 2 dBA for 1,500 simulation steps, and the prediction accuracy increased with the increments in simulation steps. In particular, noise prediction for moving machines needed more simulation steps in the prediction process to ensure that all moving machines had possibly passed through the bounded area, so the final results could be more precise and rational. In the interim, the prediction of noise exposure levels was in the range of measurement results. Indeed, it could not represent the actual circumstances in the workplace because workers were not necessarily working for the entire period in that particular area. Theoretically, the prediction indicated the risk of noise exposure for the entire period of stay in that particular area. Noise prevention is needed if the noise exposure level is over the permissible level [83].

The proposed approach requires the development of strategic noise mapping in order to obtain machine emission levels and mapping dimensions. Machine emission levels can be easily obtained from the measurements by applying the British Standards BS EN ISO 3744:2010 [81] and BS ISO 6393:2008 [82]. Also, the data can be reused for new noise mapping prediction in daily working activities. On the basis of the results from the three case studies, strategic noise maps obviously provided good visualisation to distinguish the pattern of noise propagation from machinery. It was easy to recognise the locations of stationary noise sources from the noise maps. However, the noise maps could not show the actual location of moving machines but they could map the noise polluted area to reflect the random movement of machines in that bounded area. Case studies 2 and 3 revealed the expansion of noise levels from moving machines and noise impact on the surrounding environments. The results also showed that adoption of the approximation from BS5228-1:2009 [60] for noise barriers were suitable for application in noise mapping prediction because it reduced the cost of calculation and speeded up the prediction process. The absorbent effect of noise barriers affected sound propagation from noise sources [84]. Incorporating the noise attenuation and reflection from barriers into the noise mapping enhanced the performance of noise mapping by indicating the influence of barriers on the noise propagation paths. Similarly, the contour lines, filled colours, and labels of noise levels on each contour line were crucial to illustrating the noise circumstances in the workplace.

Computers accelerated the noise mapping calculation and predicted the noise maps within a short time. Yet there are some factors that might have affected the speed of calculations in this simulation framework, such as grid refinement, number of machines, number of barriers, and the simulation steps. Higher resolution of grid data slows down the simulation process due to handling large data calculation in every simulation step. The study assumed a 1-meter interval between grid points, which was enough to handle the current noise mapping prediction with tolerable simulation speed and smooth contour lines. However, if the dimensions of the mapping area are small, then the adjustment of a smaller interval grid point is needed. This is due to the effect of size of grid refinement on data accuracy and quality of the result [85]. Likewise, increases in the number of machines, barriers, and simulation steps requires more time for completing a simulation process. Actually, it depends on the activity situation, with regard to the operating machinery and the complexity of wall barriers in a mapping area. Decision makers play an important role in the implementation of the simulation process to achieve a level of certain accuracy that they consider can reflect the real noise situation in their workplaces. Achieving a high accuracy of the prediction results requires more times to run the simulation process. Nevertheless, the application of this proposed approach is better than the conventional field measurement method because it eliminates laborious processes. It is faster, more cost-effective, and easier to apply in workplaces.

In addition, implementation of the proposed approach is important to the future development of noise mapping in the workplace. Strategic noise maps are a useful managerial tool for presenting information on current noise circumstances that can be used to predict the noise levels for future scenarios [19]. Management could predict noise levels for upcoming activities during the planning stage. They could predict a strategic noise mapping by trying different equipment and deciding which equipment is the most suitable for an activity. The information from strategic noise mapping could definitely be used for comparison with the permissible noise levels and for keeping noise levels from affecting surrounding communities. It could minimise complaints from the public and condemnation from environmental authorities. From an ergonomic standpoint, management could choose a strategic placement of new machines from strategic noise mapping. Hence, they could choose the best location for installation of new equipment such that it would not change the noise levels much in their workplaces. As mentioned in the previous section, noise mapping results are used to predict noise risk zones and reveal the risk percentage in a workplace. It is important to increase the awareness of workers by allowing them to self-perceive the risk of noise in their working environment. This is due to previous studies which showed that workers were reluctant to wear hearing protection devices (HPD) regularly and there was a low utilisation rate of hearing protection in workplaces [86, 87]. The main reasons workers disregarded the protective function of HPD was that they could not self-perceive the risks of noise and had low self-efficacy in HPD use [88]. Consequently, noise risk zones indicate potential risk areas, and workers themselves could recognise the noise risk in their working area and increase self-efficacy in HPD use.

## Conclusion

The stochastic simulation framework was successfully implemented to predict strategic noise maps and personal noise exposure levels. The present study advanced the use of the stochastic technique in noise prediction and increased the quality of noise mapping in workplaces. This framework considered the complex interaction and randomness of noise emission from machinery. The random walk approach was facilitated to simulate the random movement of machinery and workers. Data validation for the study found high accuracy of prediction results and fast production of strategic noise mapping compared with conventional field measurement methods. Hence, simulation software could be used as a managerial tool during the planning process, where it could assist in noise control and monitoring. Eventually, this technique should be widely used in current industrial practice to provide sustainable workplaces with moderate noise levels. Increasing the awareness of workers about noise problems can be done by disseminating information on strategic noise mapping and noise risk zones to the working area.
